# Editorial: Advances in Transoral Approaches for Laryngeal Cancer

**DOI:** 10.3389/fonc.2018.00455

**Published:** 2018-10-17

**Authors:** Cesare Piazza, Giorgio Peretti, Vincent Vander Poorten

**Affiliations:** ^1^Department of Otorhinolaryngology, Maxillofacial, and Thyroid Surgery, Fondazione IRCCS National Cancer Institute of Milan, University of Milan, Milan, Italy; ^2^Department of Otorhinolaryngology – Head and Neck Surgery, University of Genoa, Genoa, Italy; ^3^Department of Otorhinolaryngology – Head and Neck Surgery, University Hospital Leuven, Leuven, Belgium; ^4^Section Head and Neck Oncology, Department of Oncology, KU Leuven, Leuven, Belgium

**Keywords:** transoral access, laser surgery, laryngeal cancer, robotic surgery, minimally invasive surgery

If the reader googles words such as “transoral laser (or robotic) surgery for laryngeal cancer,” he will quickly realize how such relatively new topics are among the most actively discussed in the recent years by the international head and neck cancer community. Whether this is truly (and exclusively) the result of the publication in 1993 of Steiner's first large series of glottic tumors treated by transoral laser microsurgery (TLM) (1) and the introduction of the European Laryngological Society (ELS) classification scheme of endoscopic cordectomies in 2000 (2) as hypothesized by Mendelsohn and Remacle, can be a matter of debate. History will tell: as in all human developments, longer follow-up time and assessment by judges that are at some distance are needed to properly evaluate the historical roots of such a complex scientific, economic, and social phenomenon. However, no one can forget the value of such seminal papers and their groundbreaking effect when first published in the last century. Once the revolutionary Steiner's concept of multi-bloc trans-tumor resection of large volume laryngo-hypopharyngeal malignancies was demonstrated to not hamper any of the mainstays of oncologic radicality, given that an adequate preoperative three-dimensional (3D) model of the tumor to be removed had been fully understood, a number of “traditional” surgeons (or their younger scholars) gradually shifted their indications for management of laryngeal cancer from open to transoral approaches.

In this sense, minimally invasive surgery of the larynx essentially relies on the perfect mastering of every possible diagnostic tool available. As demonstrated by Ruytenberg et al. Magnetic Resonance Imaging has gradually emerged as the preferred method of investigation in such a field due to its superior capability of space and tissue density resolution. By designing dedicated laryngeal radiofrequency coils and applying respiratory-triggered acquisition techniques that minimize breathing and swallowing related artifacts, high-quality images (with an isotropic resolution of up to 1 mm) acquired in a few minutes will allow surgeons to obtain a set of nearly histopathologic macro-sections of the entire larynx, with marvelous understanding of the 3D extent of the lesion to be treated.

As already demonstrated by several reports, among which those first published by Blakeslee in 1984 (3) and later reproduced by Peretti et al. (4, 5), Hamzany et al. showed that an accurate pre- and intraoperative diagnostic work-up allows not only to avoid any preemptive biopsy, directly treating a given glottic lesion by a single stage TLM session, but also that it is possible to minimize the rates of over- and undertreatments in terms of depth of partial cordectomy to 10%. In particular, the routine use of intraoperative visual aids such as Narrow Band Imaging, based on the biologic endoscopy concept previously described by two of us (6), may greatly help in reducing the amount of surgical mistakes in terms of superficial positive margins, one of the most potent prognosticators in every oncologic surgical procedure, as herein demonstrated in a series of 634 patients by Fiz et al.

Even though the vast majority of papers in the literature details outcomes of primary TLM for management of naive glottic tumors,  Meulemans et al. confirm the applicability of such a therapeutic weapon even in the (much more troublesome) scenario of selected post-actinic persistent/recurrent tumors. Even though oncologic outcomes remain satisfactory, not surprisingly the Belgian Authors found a statistically significant lower local recurrence-free survival and ultimate local control rate in such a clinical setting. However, being able to ultimately preserve the larynx in 65% of their patients, the Authors definitively turned the old tenet upside down that the only possible salvage option after radiation failure would be total laryngectomy.

A comprehensive approach to the categorization of different subgroups of glottic lesions to be treated by TLM is applied by Piazza et al. rigorously assessing their dataset of 410 T1–T3 untreated patients and trying to overcome the intrinsic limitations of the contemporary edition of the TNM staging system in giving an affordable prognostication. The resulting 3D map of isoprognostic zones can effectively guide surgeons within their multidisciplinary teams in discussing the pros and cons of transoral management of a glottic tumor, balancing alternative therapeutic options in the search for the best compromise between oncologic cure and functional results.

Even though less frequently encountered and with different loco-regional patterns of spreading, even supraglottic carcinomas may be effectively managed by TLM as herein demonstrated by two independent groups of Italian and German surgeons. In fact, both Carta et al. and Ambrosch et al. report excellent oncologic outcomes in treating T1–T3 (and selected T4 lesions in the German series) with such an approach, claiming optimal vocal and swallowing outcomes that are comparatively better than those traditionally described after open-partial laryngectomies or non-surgical treatments. Interestingly, the Italian group also proposes a slight technical modification to the more advanced type of endoscopic supraglottic resection according to the ELS classification (7), by preserving the inferior third of the arytenoid cartilage and thus greatly reducing the risk of long-lasting post-operative aspiration described by others after such a procedure (8). Tumors of the posterior laryngeal compartment, i.e., involving the posterior third of the vocal cords, posterior commissure, and medial aspect of the arytenoid cartilages, have been traditionally regarded as biologically insidious and technically demanding, especially in case of superficial lesions not requiring open surgery, but still located in a difficult area to be managed by the transoral route via a standard orotracheal intubation. Mora et al. herein brilliantly introduce subglottic high frequency jet ventilation through a 4 mm laser-safe catheter as a method that allows superb control of superficial resection margins in such a rare tumor location.

One of the most limiting factors in teaching and learning TLM techniques is the contemporary relative paucity of anatomical descriptions and text books specifically focusing on structural details from the inside out. This, indeed, is definitively the most intriguing nuance represented by the work of Perotti et al. depicting in great detail the vascular anatomy of the supraglottic, glottic, and subglottic regions, with special attention to practical tips that every transoral surgeon should master in order to anticipate or treat bleeding in such procedures.

Interestingly, about 25% of papers in the present Research Topic have been devoted to discussion of transoral robotic surgery (TORS) for laryngeal cancer. A comprehensive review on the subject has been performed by Gorphe demonstrating in a balanced way the present pros and cons of such a surgical approach to the laryngeal oncologic issues. Even though comparative analysis between the “classic” TLM technique and TORS are still scant and frequently surpassed by quickly evolving robotic technology, this new approach to minimally-invasive surgery performed through natural orifices is clearly here to stay. For example, some may argue that the reported tracheostomy rate (32%) in the large series of supraglottic cancers managed by TORS from Stubbs et al. and, especially, their relatively long decannulation time (4 months) are less appealing than those reported in the major TLM series, two of which are also included in the present collection. However, as usual, direct comparisons between different series, with a different tumor mix treated in diverse health care systems, are difficult and limited in their intrinsic value. Moreover, new robots and improved anesthesiologic techniques may well change the treatment profile of these patients in the near future. The growing active interest in TORS is also evoked by Mäkitie et al. Their short paper is a purely epidemiological view on TORS in the Scandinavian countries, not addressing oncological outcomes, need for additional non-surgical treatment, or complications. The ambition of this paper is no more than an urgent invitation to summarize the need for rigorous data collection within a future clinical trial, as well as for prospectively formulating high management standards to minimize future side effects and undue sequelae. A complicating factor in interpreting the findings is that most of the centers analyzed are obviously in the beginning or the middle of their learning curve. The Authors do not provide scientific data detailing the likely (causal) relationship between the number of surgeries and outcomes, nor on the expected positive effect of their proposed further centralization (9). This criticism prompted some esteemed Editorial Board members to recommend against publication, while others heavily supported its inclusion in the present Research Topic. The final decision, as you can see, was to include such a paper as a photograph of a peculiar moment in the development of TORS in a large portion of the European Community.

In conclusion, we are strongly convinced that some of the nuances presented in this Research Topic, and definitively many others still to be developed or published, will greatly contribute in reshaping the future indications, outcomes, and limits of transoral treatments of laryngeal cancer. Collation of such different hints, suggestions, and proposals starts from the research benches, proceeds through publications and conferences, and ends up in the operatory rooms and outpatient clinics of the real world to improve overall laryngeal cancer care. We are deeply honored to be part of such a chain, and truly hope this Research Topic will fuel further interests in readers and scholars.

## Author contributions

All authors listed have made a substantial, direct and intellectual contribution to the work, and approved it for publication.

### Conflict of interest statement

The authors declare that the research was conducted in the absence of any commercial or financial relationships that could be construed as a potential conflict of interest.
